# LncRNA MBNL1‐AS1 represses cell proliferation and enhances cell apoptosis via targeting miR‐135a‐5p/PHLPP2/FOXO1 axis in bladder cancer

**DOI:** 10.1002/cam4.2684

**Published:** 2019-11-25

**Authors:** Xiaosong Wei, Xiaoming Yang, Beibei Wang, Yang Yang, Zhiwei Fang, Chengzhi Yi, Lei Shi, Dongkui Song

**Affiliations:** ^1^ Department of Urology The First Affiliated Hospital of Zhengzhou University Zhengzhou People's Republic of China; ^2^ Department of Pathology The First Affiliated Hospital of Zhengzhou University Zhengzhou People's Republic of China

**Keywords:** bladder cancer, FOXO1, MBNL1‐AS1, miR‐135a‐5p, PHLPP2

## Abstract

LncRNAs have been shown to play essential roles in bladder cancer (BC) progress. Our microarrays of clinical samples firstly screened that lncRNA muscleblind‐like 1 antisense RNA 1 (MBNL1‐AS1) was poorly expressed in BC tissues. However, its biological function in BC remains not well understood. Here we examined the clinical correlations with MBNL1‐AS1 in BC patients. Then, 5673 and T24 cell lines were employed to investigate the role of MBNL1‐AS1 in the proliferation and apoptosis of BC cells in vitro and in vivo. Furthermore, miR‐135a‐5p (miR‐135a)/PHLPP2/FOXO1 axis was focused to explore its regulatory mechanism in BC. The results showed that MBNL1‐AS1 was significantly downregulated in bladder tumor tissues, and associated with BC progression. In vitro, MBNL1‐AS1 knockdown increased the number of viable cells and bromodeoxyuridine‐positive cells, accelerated cell cycle, and dysregulated proliferative regulators (Ki67, p21, p27, and Cyclin D1) in BC cells. The apoptotic cells and the cleavages of caspase‐3/9 were reduced in MBNL1‐AS1‐silenced BC cells. Overexpression of MBNL1‐AS1 had opposite effects on BC cell proliferation and apoptosis. Moreover miR‐135a was demonstrated to interact with MBNL1‐AS1, and inhibiting miR‐135a reversed the effects of shMBNL1‐AS1 on BC cells. The downstream effectors (PHLPP2 and FOXO1) were positively regulated by MBNL1‐AS1, but negatively regulated by miR‐135a. Similar results were also observed in xenograft tumors. In conclusion, this study firstly suggests that MBNL1‐AS1 acts as a tumor suppressor of BC by targeting miR‐135a/PHLPP2/FOXO1 axis, providing a novel insight for BC diagnosis and treatment.

## Graphical abstract

(a) Muscleblind‐like 1 antisense RNA 1 (MBNL1‐AS1) suppresses bladder cancer (BC) cell proliferation, (b) MBNL1‐AS1 promotes BC cell apoptosis, and (c) MBNL1‐AS1 regulates BC progression via miR‐135a‐5p/PHLPP2/FOXO1 axis.

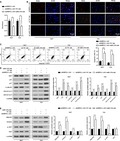

## 1 INTRODUCTION

Bladder cancer (BC) is one of the most malignant tumors of the urinary system in worldwide.^1^ Bladder urothelial carcinoma represents more than 90% in all BCs, which is a class of heterogeneous solid tumors composed of papillary tumors and solid invasive carcinomas. Once it spreads into bladder muscle layer, the mortality of patients may be reached to 85% within 2 years of diagnosis.^2^ Some advances have been exploited in the prevention and remedy of BC in recent years.^3^, ^4^, ^5^ However, the potential mechanism and effective biomarkers of BC pathological development require further investigations.

LncRNAs are recognized as a class of long noncoding RNAs approximately more than 200 nucleotides, which are closely associated with occurrences of cancers.^6^, ^7^ A growing number of evidence has demonstrated that lncRNAs, such as ZEB1‐AS1, PEG10, and UCA1, play important regulatory roles in the proliferation, migration, and invasion of BC.^8^, ^9^, ^10^ Based on our preliminary lncRNA microarrays, lncRNA muscleblind‐like 1 antisense RNA 1 (MBNL1‐AS1) level in bladder tumor tissues is decreased by 99.2 times than that in adjacent nontumor tissues. Limited studies have revealed the function of MBNL1‐AS1 in other cancers. Li et al have demonstrated that MBNL1‐AS1 exhibits an inhibitory role in the proliferation, migration, invasion, drug resistance, and sphere formation of NSCLC cells.^11^ Furthermore, MBNL1‐AS1 is also reported to inhibit the proliferation and induce the apoptosis of skeletal muscle cells.^12^ Nevertheless, its exact role and molecular mechanism in the progression of BC have not been elucidated.

Increasing evidence reveal that lncRNAs can function as competitive endogenous RNAs to regulate multiple biological processes, including cell proliferation, differentiation, apoptosis, and angiogenesis by sharing miRNAs.^13^, ^14^, ^15^ MiRNAs are endogenous single‐stranded noncoding RNAs with 22‐25 bp in length, which can negatively regulate the target gene expressions by binding with its 3′ untranslated region. Previously, miR‐135a‐5p (miR‐135a) expression has been shown to be upregulated in BC cells and tissues, and it performs as an onco‐miR by targeting PHLPP2 and FOXO1.^16^ Interestingly, in the present study, we found that MBNL1‐AS1 was directly bound with miR‐135a through bioinformatics. The PHLPP2 (pleckstrin homology domain leucine rich repeat protein phosphatase 2) and FOXO1 (forkhead box O1) have been found to act as tumor suppressors in cancer cells negatively associating with AKT signaling pathway.^17^, ^18^, ^19^, ^20^ Therefore, our current study was designed to reveal whether MBNL1‐AS1 modulated miR‐135a/PHLPP2/FOXO1 axis to control the development and progression of BC.

## 2 METHODS

### 2.1 Clinical samples and cell culture

In this experiment, 21 fresh bladder tumor and matched adjacent nontumor bladder tissue samples were obtained from The First Affiliated Hospital of Zhengzhou University. All tumor tissues were identified as urothelial cancers by pathological examinations. Tissue samples were stored in liquid nitrogen for further analysis. Patients did not receive any chemotherapy or radiotherapy before surgery. Each patient had written informed consent and this study was approved by The First Affiliated Hospital of Zhengzhou University. Human BC cell lines 5637 (Procell) and T24 (ZhongQiaoXinZhou) were cultured in RPMI‐1640 medium (31800‐022, Gibco), supplemented with 10% fetal blood serum (FBS; SH30084.03, Hyclone) in a container with 5% CO_2_ at 37°C.

### 2.2 LncRNA microarray analysis

Total RNAs were isolated from tissues using mirVana^™^ RNA Isolation Kit (AM1561, Life Technologies) and determined by NanoDrop ND‐2000 (Thermo). The integrity of RNAs was assessed using Agilent Bioanalyzer 2100 (Agilent Technologies). Then total RNAs were generated into cDNAs and hybridized onto the microarray analysis. After washing, the arrays were detected using the Agilent Scanner G2505C (Agilent Technologies). The normalized microarray data were used for further analyses. Differentially expressed lncRNAs were identified as a threshold of fold change ≥2.0 and *P* ≤ .05 with *t* test.

### 2.3 Cell transfection

Short hairpin RNA (shRNA) targeting MBNL1‐AS1 (shMBNL1‐AS1): sense 5′‐GATCCGAACGAAAGGAGCAGGGTATTTCAAGAGAATACCCTGCTCCTTTCGTTTTTTTA‐3′ and antisense 5′‐AGCTTAAAAAAACGAAAGGAGCAGGGTATTCTCTTGAAATACCCTGCTCCTTTCGTTCG‐3′; negative control shRNA (shNC): sense 5′‐GATCCCCTTCTCCGAACGTGTCACGTTTCAAGAGAACGTGACACGTTCGGAGAATTTTT‐3′ and antisense 5′‐AGCTAAAAATTCTCCGAACGTGTCACGTTCTCTTGAAACGTGACACGTTCGGAGAAGGG‐3′ were designed and synthesized. MiR‐135a inhibitor (miR‐135a inh) and negative control inhibitor, as well as miR‐135a mimics and NC mimics were purchased from JTS scientific. The overexpressed adenoviral vector of MBNL1‐AS1 (Ad‐MBNL1‐AS1) and negative control adenovirus were purchased from Wanleibio. Cell transfection was performed using the reagent Lipofectamine 2000 (11668‐019, Invitrogen) according to manufacturer's instructions. In addition, stably transfected cells were selected using G418 antibiotic (11811023, Invitrogen).

### 2.4 Tumor xenograft

Animal protocols were conducted according to the Guide for the Care and Use of Laboratory Animals, which was approved by The First Affiliated Hospital of Zhengzhou University. The BALB/c nude mice (6‐week‐old) were kept in a standard environment. 5673 cells and T24 cells stably transfected with shMBNL1‐AS1 or shNC were subcutaneously injected into the right flank of axilla, respectively. Tumor size was measured every 3 days from day 7 and calculated by the formula: length × width^2^ × 0.5. After the 19‐day injection, mice were sacrificed for further examinations, and tumor weight was measured.

### 2.5 Dual luciferase reporter assay

Bioinformatics analysis screened that miR‐135a had complementary binding sites with MBNL1‐AS1. MBNL1‐AS1 was point mutated or mismatched to evaluate the binding activity of miR‐135a following manufacturer's protocols. The wild type (WT) or mutant type (MUT) of MBNL1‐AS1 was inserted into pmirGLO vector (E133A, Promega) to construct luciferase reporter vector. The 293T cells (Procell) co‐transfected with luciferase reporter vector and miR‐135a mimics were mediated by Lipofectamine 2000. The binding activity of miR‐135a was assessed by the ratio of fly luciferase activity/renilla luciferase activity using a dual luciferase reporter kit (KGAF040, KeyGen).

### 2.6 Quantitative real‐time PCR (qRT‐PCR)

Total RNAs were extracted using RNAsimple Total RNA Kit (DP419, TIANGEN) and reverse‐transcribed into cDNA with M‐MLV reverse transcriptase (NG212, TIANGEN). qRT‐PCR was performed using SYBR Green (SY1020, Solarbio) on a real‐time PCR instrument (Exicycler96, BIONEER). The relative expressions of target genes were calculated with $2^{-\Delta \Delta C_{T}}$. U6 was identified as an internal control of miR‐135a. MBNL1‐AS1 expression level was normalized to GAPDH. The primer sequences used in this study are listed in Table 1.

**Table 1:** Primer sequences used in this study

Name	Primer	Sequence (5′‐3′)
miR‐135a	RT	GTTGGCTCTGGTGCAGGGTCCGAGGTATTCGCACCAGAGCCAACTCACAT
	Forward	GCCGTATGGCTTTTTATTCCTA
	Reverse	CTGGTGCAGGGTCCGAGGTAT
U6	RT	GTTGGCTCTGGTGCAGGGTCCGAGGTATTCGCACCAGAGCCAACAAAATATGG
	Forward	GCTTCGGCAGCACATATACT
	Reverse	GTGCAGGGTCCGAGGTATTC
MBNL1‐AS1	Forward	TGGATAAGACAGTCCCTACA
	Reverse	ATTGGATTGCTTCCCACATA
GAPDH	Forward	GACCTGACCTGCCGTCTAG
	Reverse	AGGAGTGGGTGTCGCTGT

Abbreviation: MBNL1‐AS1, muscleblind‐like 1 antisense RNA 1.

### 2.7 MTT assay

For MTT assay, cells transfected with shMBNL1‐AS1 were seeded in 96‐well plates at the density of 4 × 10^3^ per well for 0, 24, 48, 72, and 96 hours. Cells with the co‐transfection of shMBNL1‐AS1 and miR‐135a inh were cultured for 48 hours. Then cells were incubated with MTT solution for 4 hours at 37°C. Finally, the optical density was measured at 570 nm using a micro‐plate reader (ELX‐800, BIOTEK).

### 2.8 Flow cytometry

After the 48‐hour transfection, cells were collected and applied to the flow cytometry detection. The determination of cell cycle was analyzed using a Cell Cycle Analysis Kit (C1052, Beyotime) according to the standard protocol. Briefly, cells were stained with PI, mixed with RNase A at 37°C for 30 minutes, and then analyzed by flow cytometer (NovoCyte, Acea Bio). Apoptotic assay was performed with an Annexin V‐FITC Apoptosis Detection Kit (C1062, Beyotime). In brief, cells were stained with Annexin V‐FITC and PI for 20 minutes at room temperature, and detected by flow cytometer.

### 2.9 Brdu incorporation assay

Cells grown on coverslips were stained with bromodeoxyuridine (Brdu) solution for 4 hours. After fixing in paraformaldehyde, cells were blocked and incubated with Brdu antibody (66241‐1, Proteintech) following manufacturer's protocols. DAPI was used to counterstain cell nuclei. Images were observed under IX53 microscope (Olumpus) and acquired using DP73 camera (Olumpus) at 400× magnification.

### 2.10 Terminal deoxynucleotidyl transferase‐mediated dUTP nick end labeling staining

For the detection of cell apoptosis, terminal deoxynucleotidyl transferase‐mediated dUTP nick end labeling (TUNEL) staining was applied using In Situ Cell Death Detection Kit (11684795910, Roche). Cells were labeled with TUNEL at 37°C for 60 minutes in dark. Then the nuclei were stained with DAPI. The TUNEL‐positive cells were observed using BX53 microscope (Olumpus) at 400× magnification.

### 2.11 Western blot

Protein samples were extracted using RIPA lysis (R0010, Solarbio) and quantified by a BCA Protein Assay Kit (PC0020, Solarbio). For the determination of protein level, an equal volume of protein samples was loaded on SDS‐PAGE gel, and then transferred onto PVDF membrane (IPVH00010, Millipore). After blocking in fresh skim‐milk, the membranes were incubated with primary antibodies overnight at 4°C and washed in TBST. Then corresponding secondary antibodies were utilized to conjugate the primary protein targets. Finally, the protein blots were visualized with ECL solution (PE0010, Solarbio), and the optical density was analyzed using Gel‐Pro‐Analyzer (Media Cybernetics).

All antibodies used in this study are listed as follows: PHLPP2 antibody (25244‐1‐AP, Proteintech), FOXO1 antibody (18592‐1‐AP, Proteintech), AKT antibody (#4691, CST), p‐AKT antibody (#4060, CST), Ki67 antibody (A11390, ABclonal), p21 antibody (10355‐1‐AP, Proteintech), p27 antibody (25614‐1‐AP, Proteintech), Cyclin D1 antibody (A0310, ABclonal), cleaved caspase‐3 (#9661, CST), cleaved caspase‐9 (#7237, CST), GAPDH antibody (60004‐1‐Ig, Proteintech), HRP‐conjugated goat anti‐rabbit antibody (SE134, Solarbio), and HRP‐conjugated goat anti‐mouse antibody (SE131, Solarbio). GAPDH was used as internal control.

### 2.12 Immunohistochemistry

Tumor tissues were harvested, embedded in paraffin, and cut into 5‐μm slices. Then sections were dehydrated using graded ethanol and treated with antigen retrieval solution. Subsequently, the slides were incubated with specific primary antibody against Ki67 (ab15580, Abcam) overnight at 4°C, and then labeled with HRP‐conjugated goat anti‐rabbit (#31460, Thermo) for 1 hour at 37°C. The immunopositive materials were developed with DAB solution (DA1010, Solarbio) and counterstained with hematoxylin (H8070, Solarbio). Finally, the slides were observed at 400× magnification.

### 2.13 Statistical analysis

All data in this study were represented as mean ± SD and analyzed by GraphPad Prism. A paired *t* test was used to test the significance of MBNL1‐AS1 expression in the clinical samples. Other data of two groups were analyzed using an Independent‐sample *t* test. One‐way ANOVA was carried out to evaluate the comparisons among multiple groups with Bonferroni's test. The associations between MBNL1‐AS1 and tumor clinical characteristics were determined using the Fisher exact test or Pearson χ^2^. *P* < .05 was statistically significant.

## 3 RESULTS

### 3.1 MBNL1‐AS1 was downregulated in BC patients and associated with BC progression

**Figure 1:**
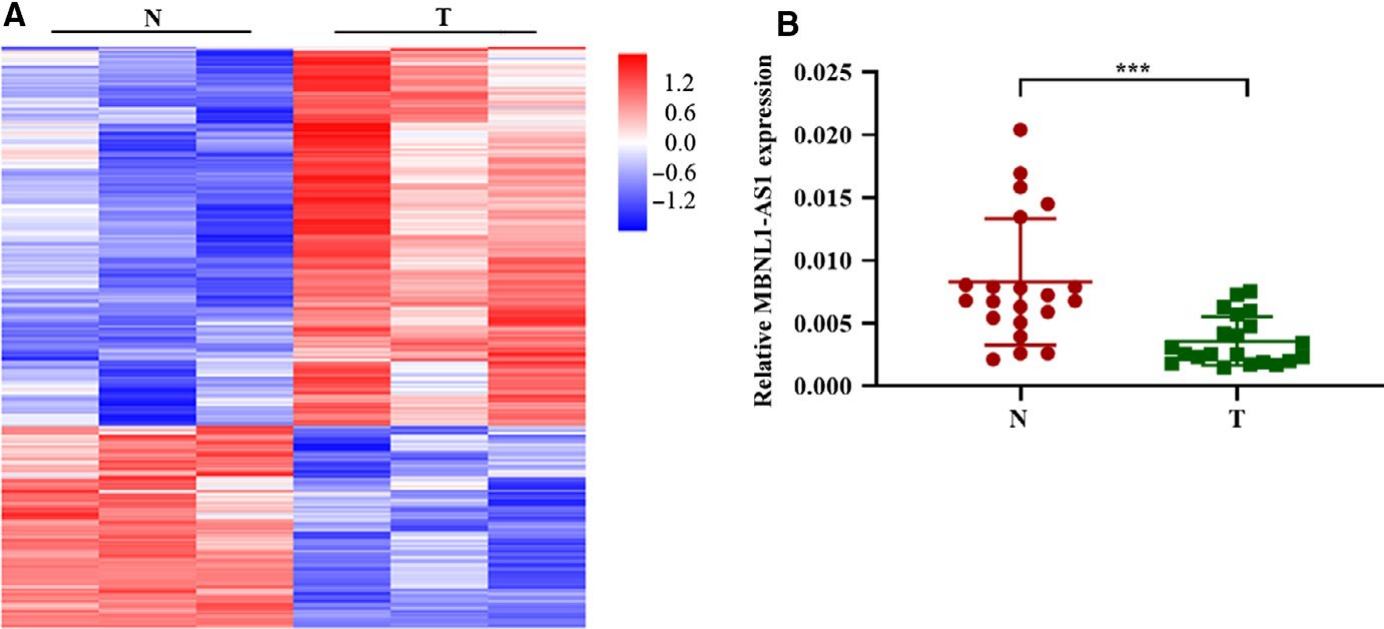
MBNL1‐AS1 was downregulated in BC patients. A, Differently expressed lncRNAs were shown with Hierarchical Clustering between tumor tissues and adjacent nontumor tissues. B, qRT‐PCR validated that lncRNA MBNL1‐AS1 was downregulated in bladder tumor tissues. BC, bladder cancer; MBNL1‐AS1, muscleblind‐like 1 antisense RNA 1; N, nontumor tissues; qRT‐PCR, quantitative real‐time PCR; T, tumor tissues. ***P < .001

The microarray analyses were performed with bladder tumor tissues and matched adjacent nontumor tissues to investigate lncRNA expression profiles of BC patients. Hierarchical clustering results showed that a total of 975 lncRNAs (636 upregulated and 339 downregulated) were differently expressed in tumors when compared to the nontumor tissues (Figure 1A). Among them, a most significant decrease of MNBL1‐AS1 expression was observed in human BC tissues. Furthermore, the qRT‐PCR analysis with clinical samples was conducted to confirm this finding from microarrays (Figure 1B).

We then further analyzed the correlations between MBNL1‐AS1 expression and clinicopathological characteristics of 60 patients with BC. The BC tumors were divided into two groups according to the expression level of MBNL1‐AS1 (high and low) based on the median value, and the correlations with clinical status of patients with BC are shown in Table 2. MBNL1‐AS1 expression level was correlated with the clinical stage, tumor size, and focal classification (Table 2). However, no significant correlations were observed between MBNL1‐AS1 and gender, age, or histopathological grade of BC patients. These results showed that MBNL1‐AS1 might be of importance in the progression of BC.

**Table 2:** Correlations between MBNL1‐AS1 expression level and clinicopathological parameters of BC patients

Clinical parameters	Cases (n = 60)	MBNL1‐AS1 expression level		*P* value
		Low	High	
Gender				.64043
Male	55	28	27	
Female	5	2	3	
Age				.59816
<60	24	13	11	
≥60	36	17	19	
T stage				.01952*
T1/T2	44	18	26	
T3/T4	16	12	4	
Histopathological grade				.31324
G1	1	0	1	
G2‐G3	59	30	29	
Tumor size				.03843*
<3cm	28	10	18	
≥3 cm	32	20	12	
Tumor number				.02846*
Unifocal	40	16	24	
Multifocal	20	14	6	

Abbreviations: BC, bladder cancer; MBNL1‐AS1, muscleblind‐like 1 antisense RNA 1.

* The *P* values had statistically significant differences (*P* < .05).

### 3.2 Knockdown of MBNL1‐AS1 enhanced the proliferation of BC cells

**Figure 2:**
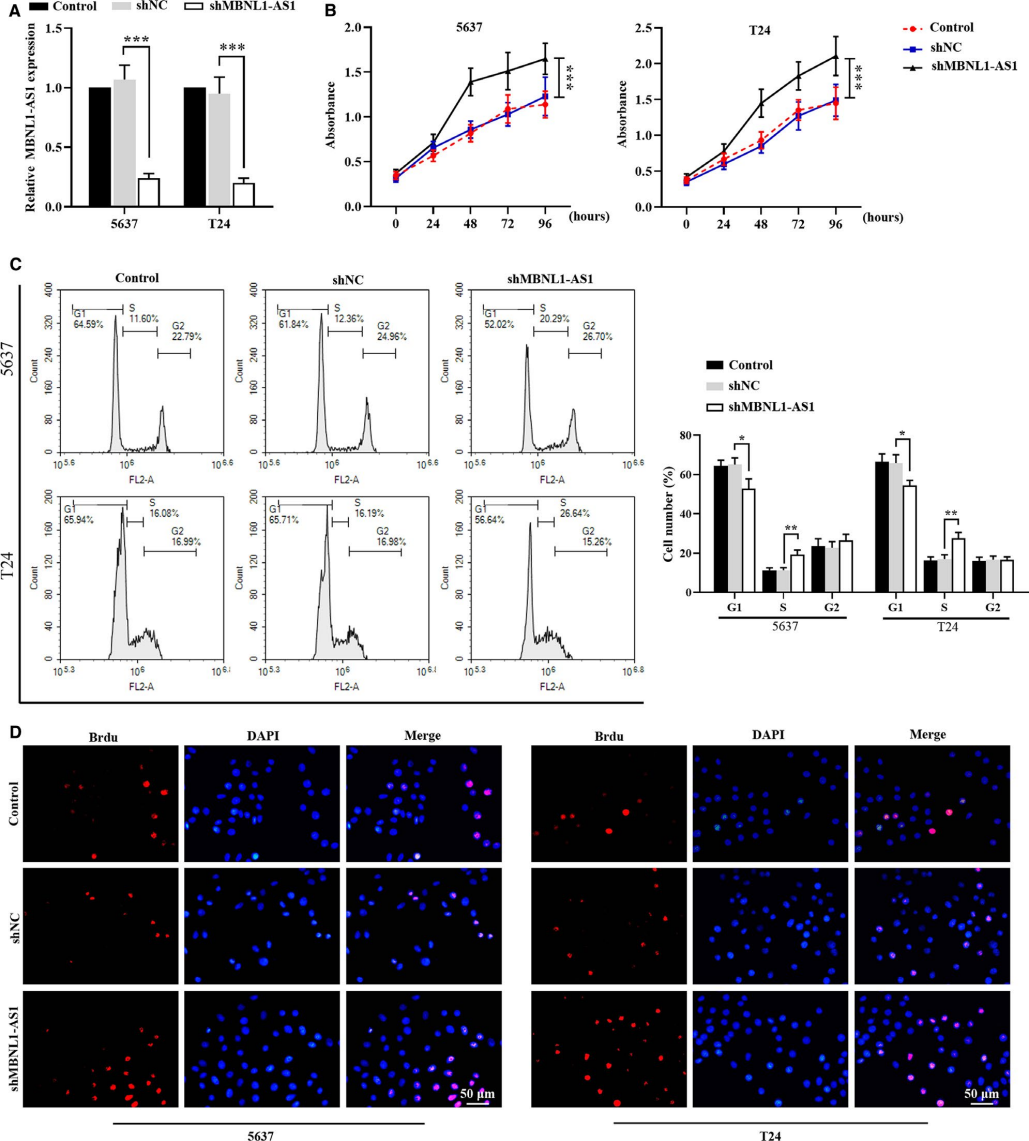
Knockdown of MBNL1‐AS1 enhanced the proliferation of BC cells. A, Relative expression of MBNL1‐AS1 in 5673 and T24 cells was detected by qRT‐PCR. B, MTT assay was applied to examine cell viability in 5673 and T24 cells. C, Cell cycle progression of 5673 and T24 cells was analyzed using flow cytometry. D, Brdu incorporation assay was used to detect the proliferation of 5637 and T24 cells. *P < .05, **P < .01, ***P < .001. BC, bladder cancer; MBNL1‐AS1, muscleblind‐like 1 antisense RNA 1; qRT‐PCR, quantitative real‐time PCR

To determine the effects of MBNL1‐AS1 on BC cell proliferation and apoptosis, human BC cell lines (5637 and T24 cells) were utilized and transfected with MBNL1‐AS1 shRNA. Expectedly, qRT‐PCR validated that the levels of MBNL1‐AS1 in both 5637 and T24 cells were significantly suppressed by its shRNA (Figure 2A). MTT assay of 5637 and T24 cells showed a remarkable increment of cell viability when MBNL1‐AS1 was silenced (Figure 2B). Furthermore, the cell cycle analysis of 5637 and T24 cells indicated that the proportion of G1 phase was significantly decreased, whereas the percentage of cell number at S phase was accumulated in MBNL1‐AS1‐knockdown cells, in comparison to cells transfected with shNC (Figure 2C). Brdu incorporation assay showed that the inhibition of MBNL1‐AS1 enhanced the DNA synthesis of 5637 and T24 cells (Figure 2D). These findings suggested that MBNL1‐AS1 knockdown promoted the proliferation, DAN synthesis, and cell cycle progression of BC cells.

### 3.3 Knockdown of MBNL1‐AS1 suppressed the apoptosis of BC cells

**Figure 3:**
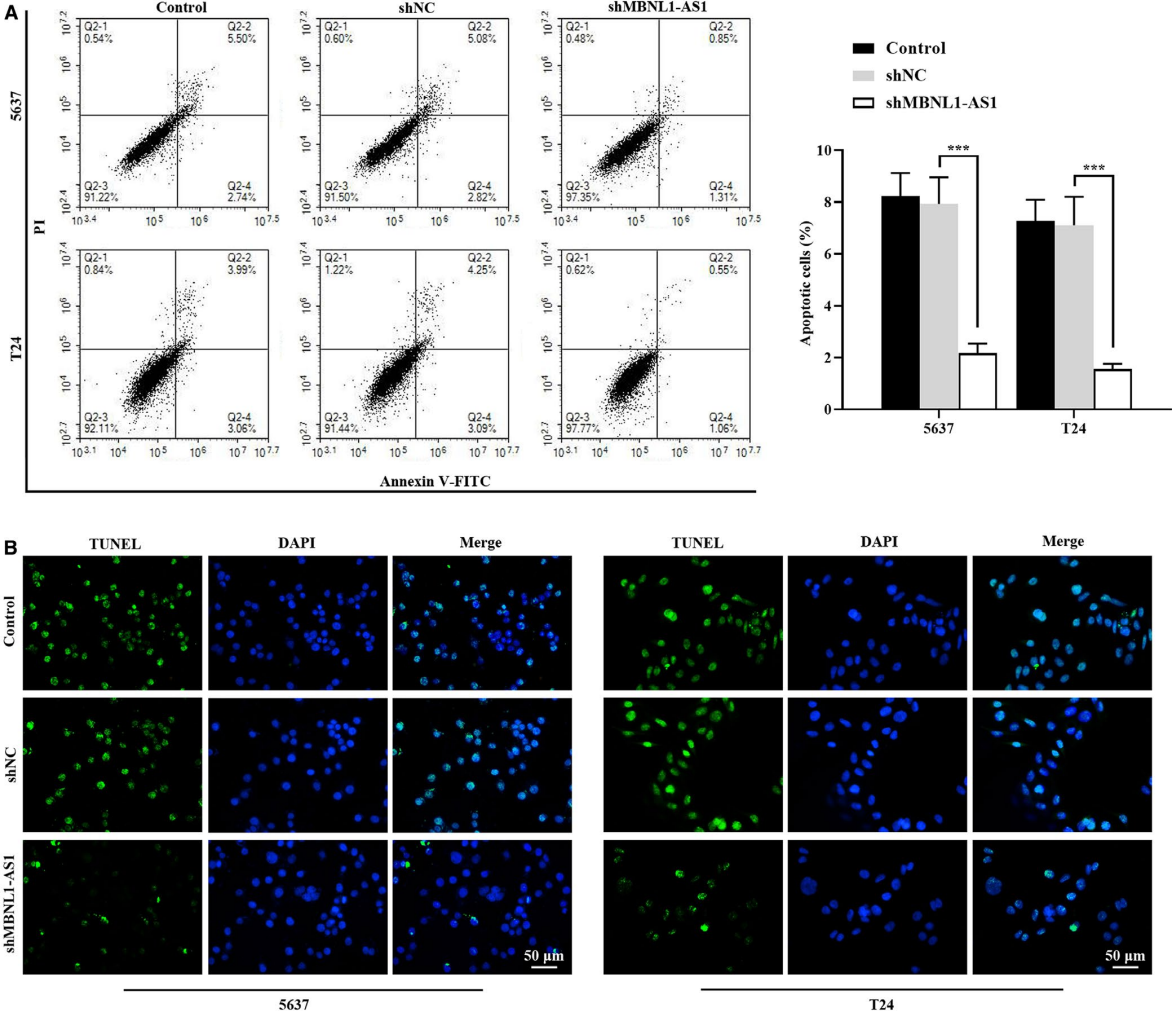
Knockdown of MBNL1‐AS1 suppressed the apoptosis of BC cells. A, Flow cytometry assay was employed to evaluate the apoptotic ratio in 5673 and T24 cells. B, The apoptotic cells were stained with TUNEL staining in 5673 and T24 cells. ***P < .001. BC, bladder cancer; MBNL1‐AS1, muscleblind‐like 1 antisense RNA 1; TUNEL, terminal deoxynucleotidyl transferase‐mediated dUTP nick end labeling

Next, we tested whether MBNL1‐AS1 regulated BC cell apoptosis. As shown in Figure 3A, flow cytometry results demonstrated that knockdown of MBNL1‐AS1 induced a dramatic decline of apoptotic ratio in 5637 and T24 cells. In addition, we also found a reduction of TUNEL‐positive cell number after inhibiting MBNL1‐AS1 (Figure 3B), indicating that MBNL1‐AS1 could promote the apoptosis of BC cells.

### 3.4 Overexpression of MBNL1‐AS1 inhibited the proliferation and induced the apoptosis of BC cells

**Figure 4:**
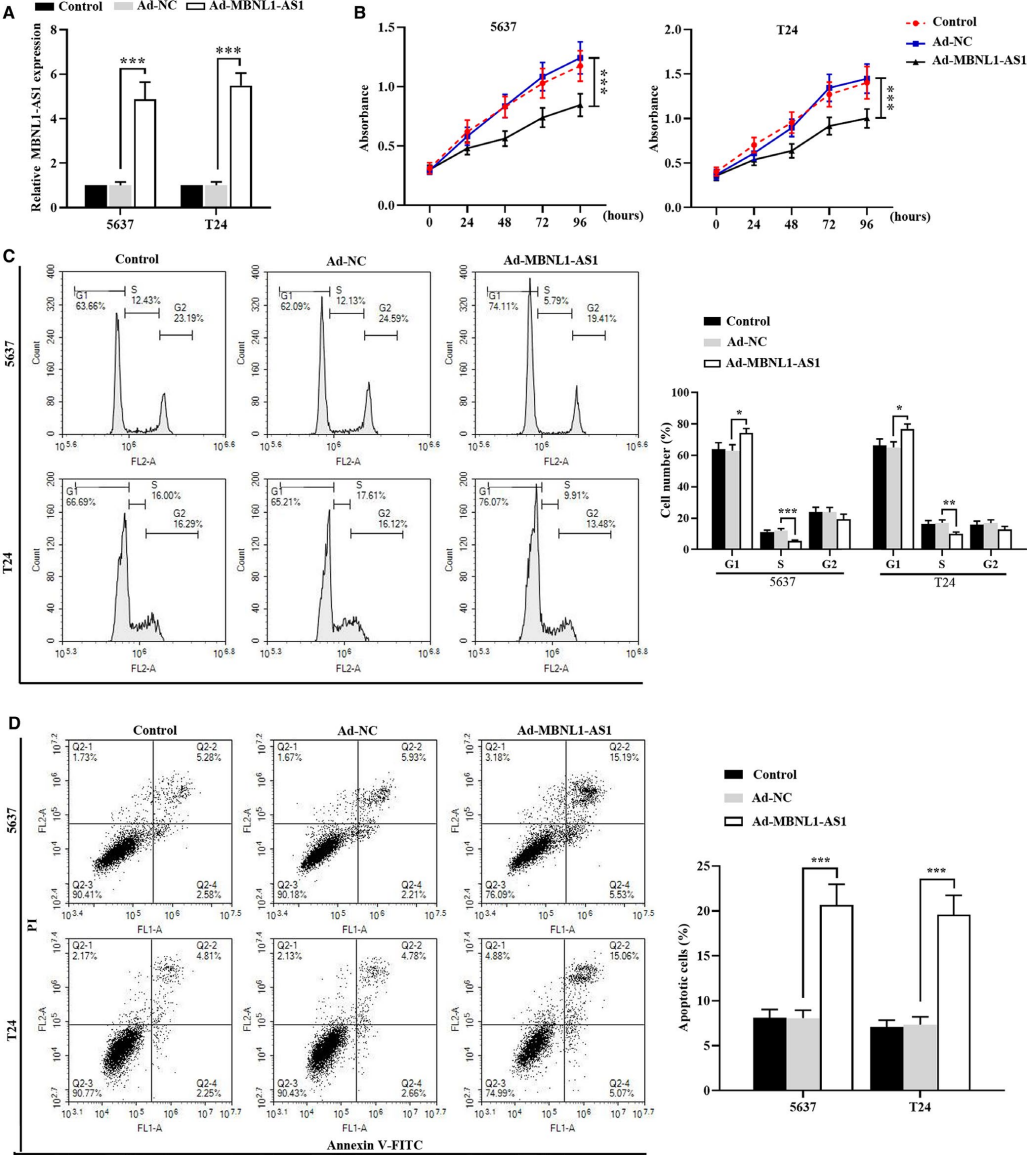
Overexpression of MBNL1‐AS1 inhibited the proliferation and induced the apoptosis of BC cells. A, Relative expression of MBNL1‐AS1 in 5637 and T24 cells was assessed by qRT‐PCR. B, The viable cells were evaluated using MTT assay in 5637 and T24 cells. C, Flow cytometry analysis was performed to determine the cell cycle distribution of 5637 and T24 cells. D, The percentage of apoptotic cells was detected by flow cytomentry in 5637 and T24 cells. *P < .05, **P < .01, ***P < .001. BC, bladder cancer; MBNL1‐AS1, muscleblind‐like 1 antisense RNA 1; qRT‐PCR, quantitative real‐time PCR

Then an adenoviral vector with MBNL1‐AS1 overexpression was further employed to determine its effects on the proliferation and apoptosis of BC cells. The relative expression of MBNL1‐AS1 was significantly increased by Ad‐MBNL1‐AS1 in BC cells in expectation (Figure 4A). Then we determined the functional effects of MBNL1‐AS1 on BC cells. As shown in Figure 4B, the number of viable cell was reduced by the ectopic expression of MBNL1‐AS1. In addition, it seemed that the cell cycle distribution was arrested at G1 phase by the overexpression of MBNL1‐AS1 (Figure 4C). Furthermore, we also observed that MBNL1‐AS1 overexpression induced significant increments of the apoptotic cells from the chart of Figure 4D. These results together indicated that the ectopic expression of MBNL1‐AS1 could suppress the progression of BC.

### 3.5 MBNL1‐AS1 directly targeted miR‐135a and positively regulated PHLPP2/FOXO1 expression

**Figure 5:**
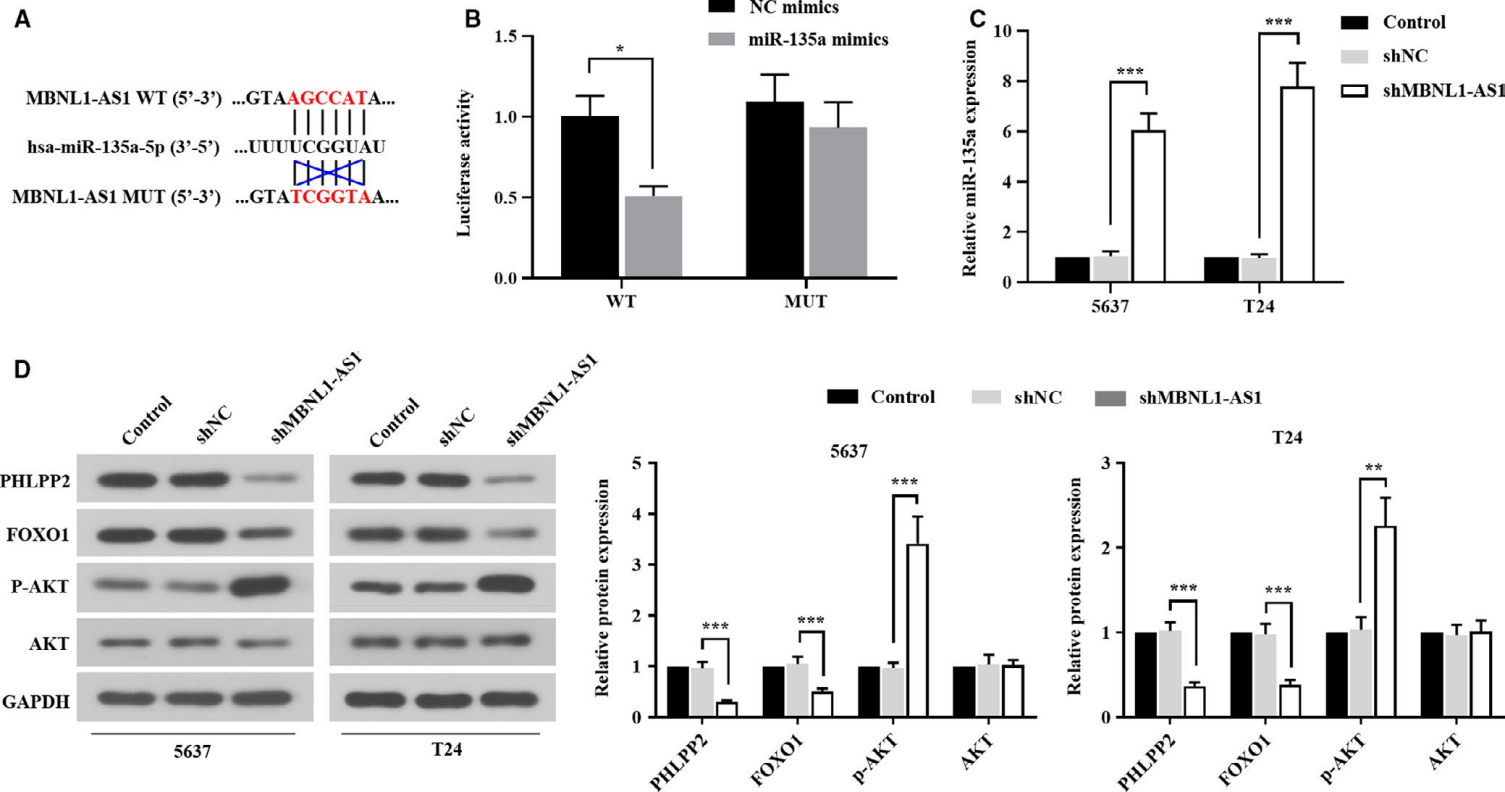
MBNL1‐AS1 was directly targeted by miR‐135a and positively regulated PHLPP2/FOXO1 expression. A, The sequences of MBNL1‐AS1 binding with miR‐135a. B, Luciferase reporter assay was carried out to evaluate the binding activity between MBNL1‐AS1 and miR‐135a. C, Relative expression of miR‐135a was determined using qRT‐PCR. D, The protein levels of PHLPP2, FOXO1, p‐AKT, and AKT were detected with western blot. *P < .05, **P < .01, ***P < .001. MBNL1‐AS1, muscleblind‐like 1 antisense RNA 1; qRT‐PCR, quantitative real‐time PCR

In order to explore the regulatory mechanism of MBNL1‐AS1 on the proliferation and apoptosis of BC cells, bioinformatics analysis predicted that miR‐135a was one of the putative targets of MBNL1‐AS1. The complementary sequences between MBNL1‐AS1 and miR‐135a, and the mutant sites of MBNL1‐AS1 were shown in Figure 5A. The dual luciferase reporter assay showed that the luciferase activity was reduced in cells co‐transfected with miR‐135a mimics and MBNL1‐AS1 WT reporter plasmid (Figure 5B). However, there was no significant difference of luciferase activity when cells were co‐transfected with MBNL1‐AS1 MUT reporter plasmid (Figure 5B). As illustrated in Figure 5C, the expression level of miR‐135a was also apparently increased by silencing MBNL1‐AS1 in 5637 and T24 cells.

Given that PHLPP2 and FOXO1 were direct targets of miR‐135a to control the malignancies of BC,^16^ we further focused on the implications of PHLPP2/FOXO1 expression in the regulation of BC progress mediated by MBNL1‐AS1. As shown in Figure 5D, western blot analysis suggested that the endogenous inhibition of MBNL1‐AS1 downregulated the expression levels of PHLPP2 and FOXO1, but upregulated the phosphorylation of AKT in 5637 and T24 cells. In addition, there was no change in total AKT protein level in neither 5637 cells nor T24 cells (Figure 5D). Collectively, these findings indicated that MBNL1‐AS1 might be a "sponge" of miR‐135a, and knockdown of MBNL1‐AS1 suppressed the expressions of PHLPP2 and FOXO1 by activating AKT signaling pathway.

### 3.6 MBNL1‐AS1 regulated the proliferation and apoptosis of BC cells via miR‐135a/PHLPP2/FOXO1 axis

**Figure 6:**
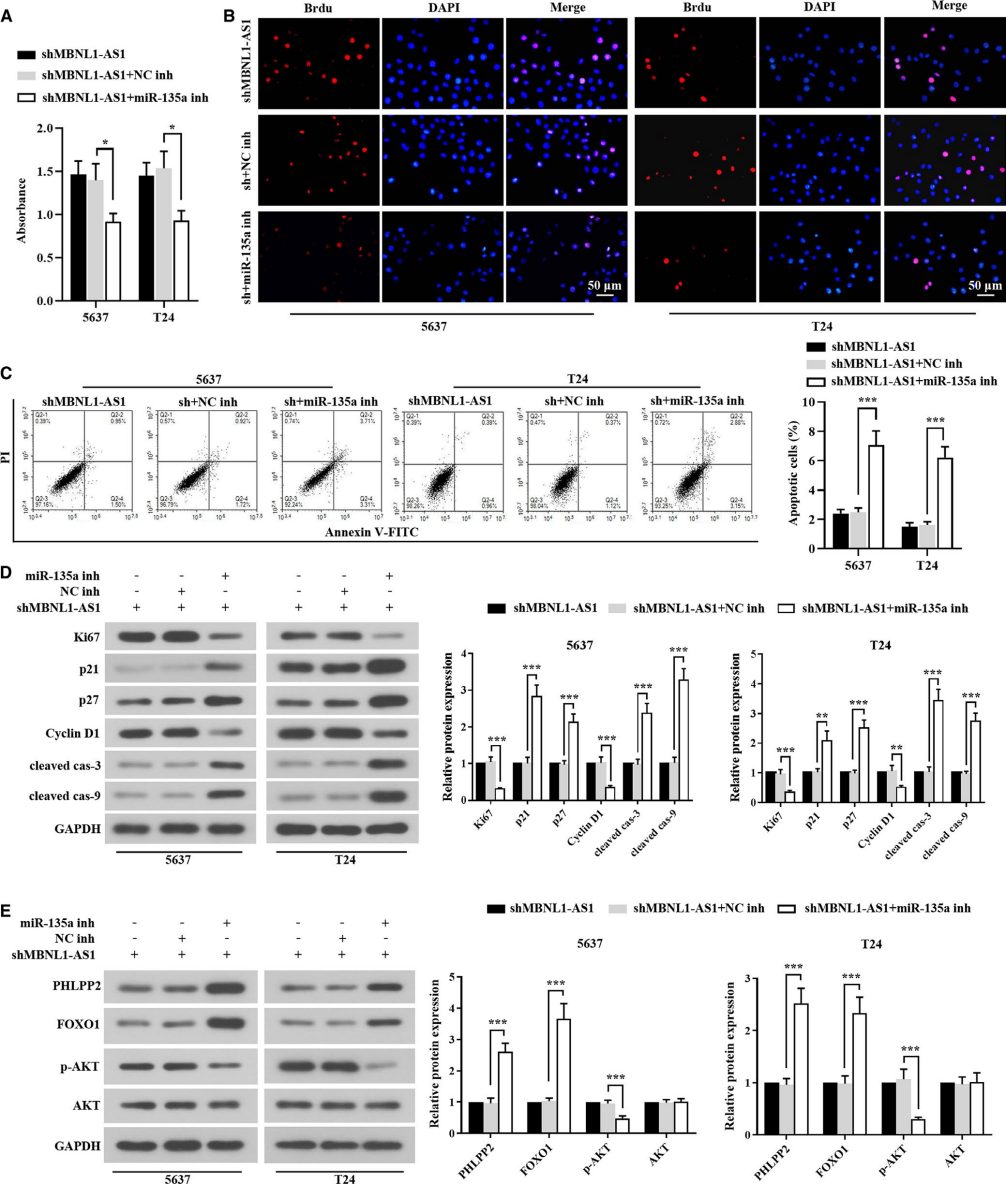
MBNL1‐AS1 regulated the proliferation and apoptosis of BC cells via miR‐135a/PHLPP2/FOXO1 axis. A, Cell viability of 5673 and T24 cells was assessed by MTT. B, The proliferative cells were detected using Brdu staining. C, Flow cytometry analysis was carried out to examine the apoptotic cells. D, Western blot analysis of cell proliferative regulators (Ki67, p21, p27, and cyclin D1) and apoptotic regulators (cleaved caspase‐3 and cleaved caspase‐9) in 5673 and T24 cells. E, Western blot analysis of PHLPP2/FOXO1 signals (PHLPP2, FOXO1, p‐AKT, and AKT) in 5673 and T24 cells. *P < .05, **P < .01, ***P < .001. BC, bladder cancer; MBNL1‐AS1, muscleblind‐like 1 antisense RNA 1

Then we used a specific inhibitor of miR‐135a to investigate its role in MBNL1‐AS1‐mediated BC cell progression. Data showed that miR‐135a inh reduced the viability of MBNL1‐AS1‐silenced 5637 and T24 cells (Figure 6A). The number of Brdu‐positive cells was also obviously suppressed by the inhibition of miR‐135a (Figure 6B). In addition, the down‐expression of miR‐135a reversed the apoptotic ratio in MBNL1‐AS1‐silenced cells (Figure 6C).

Furthermore, the effects of miR‐135a on the downstream regulator protein expressions were focused. As shown in Figure 6D, Ki67 (cell proliferation indicator) and Cyclin D1 (cell cycle marker) were significantly downregulated, whereas the cell cycle inhibitors (p21 and p27) were upregulated in 5637 and T24 cells transfected with miR‐135a inh. Similar to the alterations of cell cycle inhibitors, the decrease of miR‐135a also increased the protein expressions of cell apoptotic regulators (cleavages of caspase‐3/9). Then PHLPP2, FOXO1, and AKT protein levels were tested using western blot. As illustrated in Figure 6E, MBNL1‐AS1‐silenced 5637 and T24 cells showed dramatic increments of PHLPP2 and FOXO1 proteins when miR‐135a was suppressed. Inversely, the phosphorylation of AKT was alleviated by miR‐135a inh in 5637 and T24 cells. Taken together, the results indicated that miR‐135a played a part in the regulation of MBNL1‐AS1 on BC cell proliferation and apoptosis through PHLPP2/FOXO1.

### 3.7 Inhibition of MBNL1‐AS1 promoted the tumorigenesis of BC cells though the regulation of miR‐135a/PHLPP2/FOXO1 in vivo

**Figure 7:**
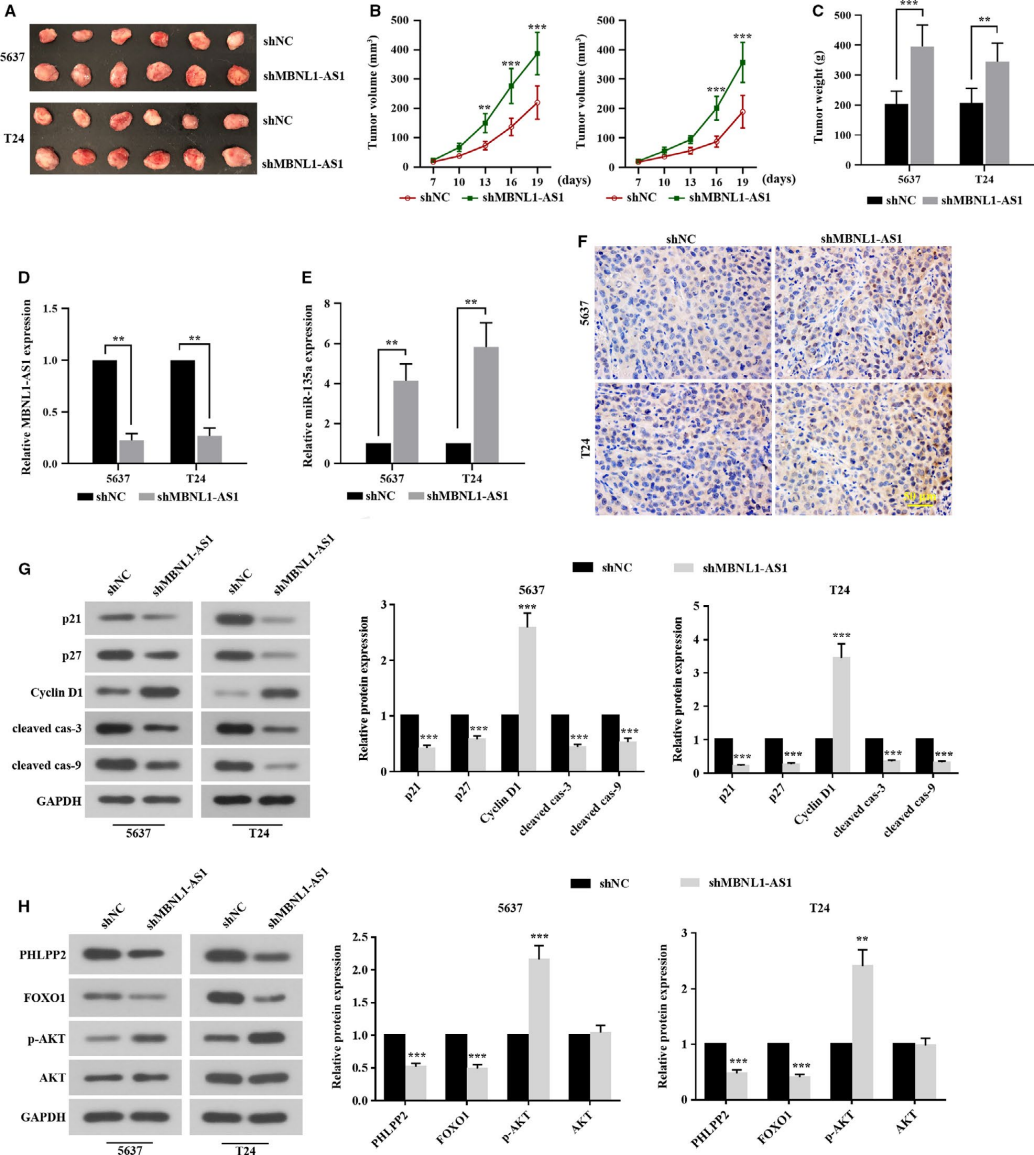
Inhibition of MBNL1‐AS1 promoted the tumorigenesis of BC cells through the regulation of miR‐135a/PHLPP2/FOXO1 in vivo. The nude mice were injected with 5673 and T24 cells stably transfected with shMBNL1‐AS1, respectively. After 19 days, mice were sacrificed. A, The tumor xenografts were collected. B, Tumor size was measured every 3 days. C, The isolated tumor was weighed at day 19. D and E, qRT‐PCR analysis of relative expression of MBNL1‐AS1 and miR‐135a. F, The immunopositive materials of Ki67 were detected using immunohistochemistry staining. G, The protein levels of cell proliferative regulators (p21, p27, and cyclin D1) and apoptotic regulators (cleaved caspase‐3 and cleaved caspase‐9) were tested by western blot. H, The protein levels of PHLPP2, FOXO1, p‐AKT, and AKT were detected using western blot. **P < .01, ***P < .001. BC, bladder cancer; MBNL1‐AS1, muscleblind‐like 1 antisense RNA 1; qRT‐PCR, quantitative real‐time PCR

Finally, xenograft mice were established to assess the effect of MBNL1‐AS1 on the tumorigenicity of BC cells in vivo. The 5637 and T24 cells stably transfected with shNC or shMBNL1‐AS1 were subcutaneously injected into nude mice. As shown in Figure 7A‐C, tumor volume and weight in shMBNL1‐AS1 group were significantly increased when compared to shNC group. We also found a decrease of MBNL1‐AS1 (Figure 7D) and an increase of miR‐135a (Figure 7E) in shMBNL1‐AS1 group. Immunohistochemistry staining also showed that the expression of Ki67 was higher in shMBNL1‐AS1 group than that in shNC group (Figure 7F). Furthermore, MBNL1‐AS1 silencing dramatically induced the upregulation of Cyclin D1, and the downregulations of p21, p27 and cleavages of caspase‐3/9. As well, PHLPP2 and FOXO1 protein was decreased, but p‐AKT was increased when MBNL1‐AS1 was knockdown. The in vivo results confirmed that MBNL1‐AS1 could block tumor formation of BC by decreasing miR‐135a and inducing PHLPP2/FOXO1.

## 4 DISCUSSION

BC is a most common malignant tumor with high mortality, which attracts great attentions on studying its roles in biological processes and molecular mechanisms. Herein, for the first time, we found that lncRNA MBNL1‐AS1 was downregulated in BC tissues in comparison to adjacent normal tissues. MBNL1‐AS1 was demonstrated to suppress the proliferation and enhance the apoptosis of BC cells in vitro and in vivo by targeting miR‐135a and positively regulating PHLPP2/FOXO1 expression. Furthermore, miR‐135a inh negatively induced the abnormal proliferation, apoptosis, and protein expressions of PHLPP2, FOXO1, and p‐AKT in MBNL1‐AS1 silencing BC cells, suggesting the novel MBNL1‐AS1/miR‐135a/PHLPP2/FOXO1 regulatory network in controlling the progression of BC mediated by the AKT signaling pathway.

Accumulating studies showed that the dysregulations of lncRNAs might be of importance for the malignancies of various cancers, such as BC. For example, lncRNA UCA1 was reported to promote the mitochondrial function and viability of BC cells.^15^ LncRNA PEG10 interacted with miR‐29b to enhance BC cell proliferation, migration and invasion.^9^ In addition, Qiu et al demonstrated that lncRNA MIR503HG acted as a tumor suppressor, which inhibited cell proliferation, metastasis and epithelial‐mesenchymal transition process in BC.^21^ It was described that MBNL1‐AS1 was a novel lncRNA to potentially predict the prognosis of gastric cancer.^22^ Li et al showed that the expression of MBNL1‐AS1 in skeletal muscle cells of mice might reduce the proliferation and induce the apoptosis.^12^ Moreover MBNL1‐AS1 with poor expression in NSCLC had an inhibitory role in the progression and drug resistance of NSCLC.^11^, ^23^ However, the role of MBNL1‐AS1 in BC progression was unclear. For the first time, MBNL1‐AS1 was screened to be down‐expressed from lncRNAs microarray database in BC patients' tumor tissues, which was further validated by qRT‐PCR analysis with tumor samples. Results showed that knockdown of MBNL1‐AS1 promoted cell proliferation and inhibited cell apoptosis of BC in vitro, but its overexpression attenuated the malignant phenotypes of BC. Altogether, these results indicated that MBNL1‐AS1 might exert suppression effects on BC cell proliferation and apoptosis.

It was widely proved that lncRNAs regulated cell biological properties through interacting with miRNAs. A recent study from Peng et al showed that lncRNA BLACAT1 was high‐expressed in human hepatocellular carcinoma, and its downregulation suppressed the proliferation and invasion by competing against miR‐485‐5p.^24^ As a critical member of miR‐135 family, miR‐135a had been investigated in multiple cancers, and its aberrant expression might be implicated in the development and progression of tumors. For instance, Zhang et al demonstrated that miR‐135a could accelerate cell growth and proliferation of lung cancer, but repress cell apoptosis.^25^ In colon adenocarcinoma, miR‐135a was suggested to be a "sponge" of lncRNA FOXD3‐AS1 to regulate cell proliferation, migration, invasion, and apoptosis by targeting SIRT1.^26^ In addition, several studies have also revealed that the positive effects of miR‐135a on BC cell proliferation, epithelial‐mesenchymal transition, migration and invasion.^16^, ^27^ However, whether miR‐135a participated in the regulation of MBNL1‐AS1 in BC progression remains unclear. We found that miR‐135a was a direct target of MBNL1‐AS1 and negatively regulated by MBNL1‐AS1. Importantly, miR‐135a reversed the effects of MBNL1‐AS1 on BC cell proliferation and apoptosis. These results suggested that MBNL1‐AS1 protected against the malignant phenotypes of BC through the suppression of miR‐135a.

Growing evidence showed that the PHLPP family members could suppress the tumorigenesis, because of their involvements in the blockage of growth factor‐induced signaling pathway by the attenuation of AKT signaling.^20^, ^28^ FOXO1 was also highlighted to be a downstream target of AKT signaling to suppress tumor development.^18^, ^19^, ^29^ Huang et al reported that PHLPP2 displayed an important effect on the invasiveness of BC cells by the regulation of lncRNA MEG3/miR‐27a axis.^17^ Evidence by Jiang et al demonstrated that FOXO1 mediated the role of miR‐145 in BC.^18^ In particular, it was apparently suggested that PHLPP2 and FOXO1 were direct targets of miR‐135a to involve in modulating BC cell proliferation.^16^ Our study showed that PHLPP2 and FOXO1 expressions were positively regulated by MBNL1‐AS1, but negatively regulated by miR‐135a. However, the activation of AKT signaling pathway by MBNL1‐AS1 silence was blocked by the inhibition of miR‐135a. Collectively, the current work confirmed that MBNL1‐AS1 regulate miR‐135a/PHLPP2/FOXO1 axis to control the progression of BC.

## 5 CONCLUSIONS

In summary, to the best of our knowledge, it is the first work to demonstrate that MBNL1‐AS1 is downregulated in BC, and MBNL1‐AS1 plays a critical role in reducing cell proliferation and enhancing cell apoptosis of BC. MBNL1‐AS1 serves as a "sponge" of miR‐135a to attenuate the regulation of PHLPP2/FOXO1, thus inhibiting BC malignancies. These findings together indicate that MBNL1‐AS1 may be a novel tumor suppressor of BC, which provides a molecular basis for BC prognosis and treatment.

## CONFLICT OF INTEREST

The authors declare no conflict of interest.
